# Efficacy and Safety of Ropivacaine Addition to Intrathecal Morphine for Pain Management in Intractable Cancer

**DOI:** 10.1155/2015/439014

**Published:** 2015-10-18

**Authors:** Ying Huang, Xihan Li, Tong Zhu, Jian Lin, Gaojian Tao

**Affiliations:** ^1^Department of Pain, Affiliated Drum Tower Hospital, Medical School of Nanjing University, Nanjing, Jiangsu 210008, China; ^2^State Key Laboratory of Pharmaceutical Biotechnology, School of Life Sciences, Nanjing University, Nanjing, Jiangsu 210093, China

## Abstract

*Objective*. Although intrathecal drug infusion has been commonly adopted for terminal cancer pain relief, its adverse effects have made many clinicians reluctant to employ it for intractable cancer pain. The objective of this study is to compare the efficacy and security of an intrathecal continuous infusion of morphine and ropivacaine versus intrathecal morphine alone for cancer pain. *Methods*. Thirty-six cancer patients received either a continuous morphine (*n* = 19) or morphine and ropivacaine (*n* = 17) infusion using an intrathecal catheter through a subcutaneous port. Numerical Rating Scale (NRS) scores and the Barthel Index were analyzed. Adverse effects and complications on postoperative days 1, 3, 7, and 15 were also analyzed. *Results*. All patients experienced pain relief. Compared to those who received morphine alone, patients receiving morphine and ropivacaine had significantly lower postoperative morphine requirements and higher Barthel Index scores on the 15th postsurgical day (*P* < 0.05). Patients receiving morphine and ropivacaine had lower NRS scores than patients receiving morphine alone on postoperative days 1, 3, 7, and 15 (*P* < 0.05). Negative postsurgical effects were similar in both groups. *Conclusions*. Morphine and ropivacaine administration through intrathecal access ports is efficacious and safe and significantly improves quality of life.

## 1. Introduction

Despite guidelines and recommendations, many patients with cancer still have inadequate pain control [1]. Many patients experience immense pain prior to their death. However, the intravenous or oral dosage of opioids for pain control results in unacceptable sedation [2]. Intrathecal therapy is advocated for patients with severe cancer pain [3].

Intrathecal injection of opioids has already been successfully utilized as a front-line treatment for cancer pain refractory to traditional treatment [4]. Intrathecal morphine has been found to be nonsedative or minimally sedative in multiple studies without the negative effects of parenteral or oral opioids [5]. Cancer pain refers to symptoms resulting from inflammation, compression, and neurological and ischemic damage at various sites [6]. According to the clinical symptoms reported, many patients who have somatic pain also have neuropathic pain. Although opioids appear to be effective in overall pain control, neuropathic pain resulting from major dysfunction of the somatosensory system [7] may be less likely to respond to opioid therapy [8]. In these conditions, opioid doses are continually increased [9]. However, increased opioid doses are associated with unsatisfactory negative effects. Therefore, the combination of opioids with other drugs, such as local anesthetics, must be considered.

Morphine exerts effects against pain by binding to the *μ*, *δ*, and *κ* opiate receptors [10], which stimulates potassium influx, giving rise to postsynaptic neuron membrane hyperpolarization in the dorsal horn of the spinal cord [11]. Voltage-sensitive calcium influx is decreased, thereby decreasing the neurotransmitter release from presynaptic terminals [12]. Ropivacaine, an amino-amide local anesthetic, blocks the generation and conduction of nerve impulses through blockade of sodium influx [13]. Studies have indicated that ropivacaine promotes the effects of intrathecal opioids [14]. Despite existing documentation and studies for the usage of intrathecal ropivacaine mixed with morphine [15], this treatment is still controversial.

Few studies have utilized an intrathecal continuous injection of ropivacaine with morphine to treat cancer pain [16]. The objective of this study was to determine the efficacy and safety of continuous ropivacaine and morphine injection using intrathecal access ports in patients suffering from cancer pain refractory to traditional treatment modalities.

## 2. Materials and Methods

### 2.1. Patient Selection

This was a double-blind, random clinical study conducted at our hospital. The institutional ethics committee was used to assign patients and provide advice regarding ethical issues. The study was approved by the institutional review board, which also confirmed informed consent by the patients. This study included thirty-six terminal cancer patients between November 2010 and September 2013 (Table 1). Eligible patients were randomly allocated to receive 1 of 2 intrathecal treatments. Randomization numbers were generated using an automated and validated system to assign treatment arms, and the assignments were concealed from patients and investigators. Constant morphine injection was given to group M patients through intrathecal access for pain administration, whereas constant morphine and ropivacaine injection was given to group R patients. All 36 patients of the study had received treatment for pain that had proven refractory to traditional therapies such as transcutaneous electrical nerve stimulation, physiotherapy, pharmacotherapy (anti-inflammatory drugs, nonsteroidal drugs, tricyclic antidepressants, oral/transdermal opioids, anticonvulsants, and antispasmodics), and psychotherapy. Patients were chosen prior to catheter placement according to the following inclusion criteria: intractable cancer pain unmanaged by high dosage of oral or parenteral analgesics, intolerability to surgery for pain, or general failure to relieve pain; emotional stability of the patient and family members; and presence of an accountable and competent care provider [2]. The patients were psychiatrically evaluated before the initial port implant and no progressive psychiatric disease was found.

### 2.2. Procedure

All 36 patients were at the hospital receiving systemic treatment. They were told about the availability of an intrathecal catheter for pain management (with dosages administered by a responsible and capable caregiver) days ahead of the catheter implantation. In addition to verbal instruction, they were presented with the intrathecal catheter and port system and informed of the implantation treatment in written form, particularly with regard to the advantages and potential side effects of intrathecal therapy. They were also advised in writing of complications and related blood tests, particularly coagulation screening, C-reactive protein and white cell count. All instructions were performed before catheter placement. Clindamycin (600 mg) was administered intravenously 2 h before catheter insertion for antibiotic prophylaxis.

Intrathecal catheterization combined with implantation of a subcutaneous infusion port (Celsite, B. Braun, France) was conducted in the sterile condition in an operating room. The patient was put in the lateral decubitus position and then draped in a sterile fashion. Lumbar puncture was performed at the interspace between L2 and S1 using an 18-gauge Tuohy needle. A 20-gauge intrathecal catheter was introduced through the Tuohy needle. The catheter was then inserted into the subarachnoid space. The catheter location was confirmed by ensuring that aspiration of cerebrospinal fluid was possible. Approximately 15–30 cm of the catheter was introduced intrathecally according to the location of the pain under fluoroscopic guidance. The catheter was moved through the subcutaneous tissue between the lumbar incisions and port pocket and then attached to the port. The port pocket was established through a bone structure (the base of the ribs was usually chosen). The port was placed into a subcutaneous pocket organized in the chosen area and attached to the fascia. Every patient underwent the procedure with no complications. Access to the port was via percutaneous injection using a special noncoring needle that was connected to a patient-controlled analgesia (PCA) device with a total volume of 100 mL and a rate of 0.5 mL/h. The original intrathecal morphine dosage was obtained from the preoperative morphine injection using an oral-intrathecal ratio of 300 : 1 [17]. The starting dose of ropivacaine was 0.375 mg/mL, and the total dose per day was 4.5 mg. The dose was titrated every 24 h until pain was reduced or therapy-limiting side effects were identified. One day before leaving hospital, the constant intrathecal dosage was selected to be the optimal dosage for placing intrathecal port. The drug container was changed on a weekly basis. The port needle was exchanged on a monthly basis.

### 2.3. Statistical Analysis

We analyzed the demographic statistics (e.g., sex and age) of the patients, cancer categories, location of pain, morphine dosage history, use of oral medications, and pain intensity before implantation of the port as reported using the Numerical Rating Scale (NRS) in which 0 means no pain and 10 means the greatest pain. The NRS score and intrathecal morphine and ropivacaine consumption on postsurgical days 1, 3, 7, and 15 were evaluated. The Barthel Index, a disability scale with scores from 0 (completely dependent) to 100 (completely independent), was used to evaluate the functional status of the patients. Data regarding technical aspects of the procedure (such as catheter tip location, injection interspace, and device-related complications) and nonpharmacological methods used to reduce cancer pain were obtained. The day before hospital discharge, the use of extrasystemic opioids and adjuvants was also recorded.

The data were analyzed using SPSS 17.0 software. The paired-sample *t*-test and two-way ANOVA were used for data comparisons. *P* < 0.05 was defined as significant.

## 3. Results

Thirty-six patients completed the trial: 17 in group R and 19 in group M. In group M, 11 male and 8 female patients were studied, with an average age of 64.0 years (40–85 years). In group R, 14 male and 3 female patients were studied, with an average age of 62.6 years (49–85 years). In group M, nociceptive pain was present in 11 patients, whereas a mixture of neuropathic-nociceptive pain was present in 8 patients. In group R, nociceptive pain was present in 7 patients, whereas mixed neuropathic-nociceptive pain was present in 10 patients. Physicians were asked to rate the pain characteristics based upon complaints from patients, with nociceptive pain defined as “aching, squeezing, or pressure-like sensations”; neuropathic pain was characterized as “tingling, burning, or electrical” sensations. The mean duration of hospital care was 12 days for patients in group M and 15 days for those in group R.

The doses of preoperative systemic opioids (intravenous and oral oxycodone, intravenous morphine, and transdermal fentanyl) were summarized as the oral morphine equivalent dose. Additional usage of opioid treatment was also documented to summarize gross opioid usage on a daily basis and presented as the variation in preoperative opioid dosage. The preoperative oral morphine consumption was between 45 and 600 mg/day (mean: 200.8 mg/day) in group M. The preoperative oral analgesic consumption was between 6 and 750 mg/day (mean: 223.7 mg/day) in group R. The mean preoperative doses of oral morphine in groups M and R were similar (*P* = 0.688).

The daily dosage of intrathecal morphine and ropivacaine was adjusted at a certain time based on the pain degree and the bolus dosage required in the last 24 h. A significant increase was observed in intrathecal morphine administration on postoperative days 3, 7, and 15 in comparison with day 1. In group M, the mean doses of intrathecal morphine on postoperative days 1, 3, 7, and 15 were 0.67 ± 0.48 mg, 1.02 ± 0.64 mg, 1.44 ± 0.86 mg, and 2.36 ± 1.56 mg per day, respectively. In group R, the mean doses on postoperative days 1, 3, 7, and 15 were 0.75 ± 0.66 mg, 0.93 ± 0.80 mg, 1.09 ± 0.99 mg, and 1.23 ± 1.10 mg per day, respectively. The mean doses of intrathecal morphine on postoperative days 1, 3, and 7 in groups M and R were similar (*P* = 0.688, *P* = 0.697, and *P* = 0.207, resp.). However, the mean dose of intrathecal morphine on postoperative day 15 in group R was significantly lower than that in group M (*P* = 0.005). In group R, the mean doses of intrathecal ropivacaine on postoperative days 1, 3, 7, and 15 were 4.5 mg, 6.10 ± 2.10 mg, 6.96 ± 2.48 mg, and 8.71 ± 6.54 mg per day, respectively. Dose escalation was guided by the clinical response. No patient received oral opioid supplementation for 15 days after the surgery.

**Figure 1:**
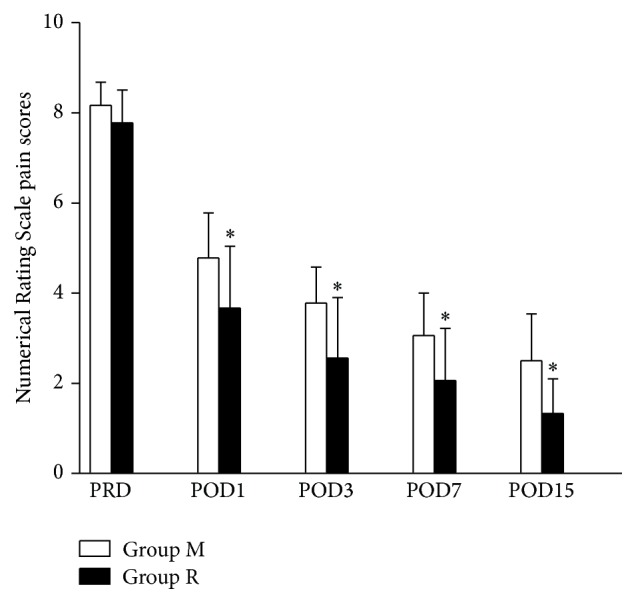
Numerical Rating Scale pain scores on pre- and postoperative days. Data are expressed as mean ± SD. ^*∗*^ *P* < 0.01 compared to the group treated with morphine alone. PRD, preoperative day; POD, postoperative day.

The average NRS scores for the 19 patients in group M on the preoperative day and postoperative days 1, 3, 7, and 15 were 8.17 ± 0.51, 4.78 ± 1.0, 3.78 ± 0.80, 3.06 ± 0.94, and 2.50 ± 1.04, respectively. The average NRS scores for the 17 patients in group R on the preoperative day and postoperative days 1, 3, 7, and 15 were 7.78 ± 0.73, 3.67 ± 1.37, 2.56 ± 1.34, 2.06 ± 1.16, and 1.33 ± 0.77, respectively. The average NRS scores on the preoperative days were similar in groups M and R (*P* = 0.069). The average NRS scores on the postoperative days 1, 3, 7, and 15 decreased gradually in both groups M and R (*F* = 40.26, *P* < 0.001 and *F* = 30.62, *P* < 0.001). However, the average NRS scores on postoperative days 1, 3, 7, and 15 were statistically lower in group R than in group M (*F* = 37.38, *P* < 0.001) (Figure 1).

**Figure 2:**
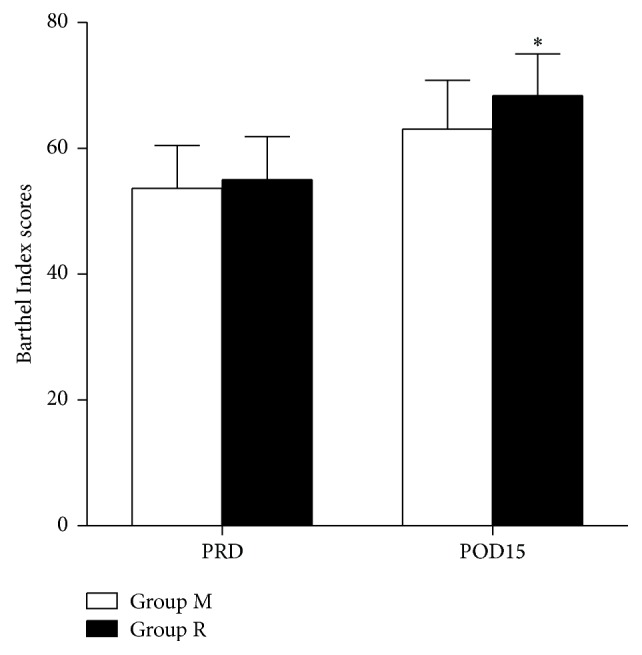
Barthel Index scores on pre- and postoperative days. Data are expressed as mean ± SD. ^*∗*^ *P* < 0.05 compared to the group treated with morphine alone. PRD, preoperative day. POD, postoperative day.

All patients in group M and group R reported an improved quality of life. The Barthel Index scores on the preoperative day and the 15th postoperative day were 53.61 ± 6.82 and 63.06 ± 7.70, respectively, in group M. The Barthel Index scores on the preoperative day and the 15th postoperative day were 55.0 ± 6.86 and 68.33 ± 6.64, respectively, in group R. The Barthel Index score on the preoperative day was similar in groups M and R (*P* = 0.472). However, the Barthel Index score on the 15th postoperative day was higher in group R than in group M (*P* = 0.017) (Figure 2).

Most intrathecal catheters were implanted at the L2-3 interspace in both group M (72.2%) and group R (61.1%), and most of the catheters reached T10 in both group M (55.6%) and group R (44.4%). Intrathecal morphine and ropivacaine presented few complications or adverse effects. No patient experienced respiratory depression. In group M, one patient experienced transient urinary retention and three experienced nausea and vomiting. In group R, one patient experienced transient urinary retention, one constipation, and one nausea and vomiting. The adverse effects were managed with conservative therapies. Urinary retention was controlled through transient urinary catheterization. No complications of intrathecal drug delivery were noted. No surgical complications were found, and meningitis and infection were not observed. Regional headaches were also not observed. Ropivacaine was added to the opioid injection without any significant dermal numbness or decreased sensation. There were no complications or adverse effects serious enough to require intrathecal port removal or treatment changes.

## 4. Discussion

The first study of intrathecal morphine in a cancer patient was performed by Tung et al. in 1980 [18]. The first study of intrathecal ropivacaine in a cancer patient was reported in 1998 [19]. The present findings are in agreement with existing studies demonstrating a decrease in pain upon intrathecal drug delivery. Our findings illustrate that administration of ropivacaine and intrathecal morphine through an indwell injection port is both efficacious and safe for severe cancer pain. Simultaneously, this study demonstrated a larger decrease in NRS rates using intrathecal morphine and ropivacaine compared to intrathecal morphine alone. Our findings demonstrate that intrathecal ropivacaine combined with morphine is safe; adverse effects were mild and rare.

The mechanism of action of intrathecal opiates is under debate. As an opiate receptor agonist, morphine blocks the activity of some cells in the substantia gelatinosa of the medullary dorsal horn [20]. As a result, intrathecal narcotic management decreases cutaneous pain transmission at the dorsal horn and transmission of nociceptive impulses through ascending pathways [21]. Several studies have found that intrathecal morphine management is an efficient approach for analgesia in humans and animals [22]. However, studies have large increases in the required dosage of intrathecal morphine with increasing cancer pain on postoperative days [23]. Among patients with insufficient pain management on intrathecal morphine, it is common to increase the opioid dosage for optimization of pain management. However, such increases are associated with adverse effects. Our study showed that the mean dose of intrathecal morphine on postoperative day 15 in patients receiving both morphine and ropivacaine was significantly lower than that in patients treated with morphine alone.

The combined intrathecal treatment is useful when insufficient pain reduction is realized with intrathecal monotherapy, especially in neuropathic pain patients. Different pain statuses have various potential mechanisms; as a result, a medication with a particular mechanism tends to only be efficient for one or two specific pain conditions. Thus, combining agents tends to be useful to treat multiple aspects of the pain, generating synergistic efficacy for pain management. Intrathecal morphine targets medullary opioid receptors, whereas intrathecal ropivacaine functions at the dorsal root and the nerve roots to promote pain management. Ropivacaine reversibly blocks sodium influx, thereby hindering pain signal transmission through A*δ* and C fibers [6]. Ropivacaine may increase opioid efficacy synergetically: (1) decreasing voltage-sensitive calcium influx, thus promoting the opioid-mediated presynaptic inhibition of neurotransmitter release from terminals of A*δ* and C fibers [2, 14] by decreasing the conformational transformation of medullary opioid receptors (*μ*, *δ*, and *κ* receptors) [24]. Our findings show higher benefit for pain management and quality of life for inpatients with cancer pain when using the combined treatment. Based on our analysis, the recommended daily dosage of intrathecal ropivacaine to realize pain control was 4.5–15.3 mg. Because we could reduce pain with a low dose of ropivacaine, the observed pain control may reflect synergy between ropivacaine and opioids. To our knowledge, this is the only study with a standard, quantitative assessment of changes in the NRS score and quality of life when the combination of intrathecal morphine and ropivacaine is used to reduce cancer pain. In our study, the average NRS scores on postoperative days 1, 3, 7, and 15 were significantly lower in patients treated with ropivacaine and morphine than in those treated with morphine alone. The Barthel Index ratings indicate that the quality of life on the 15th postoperative day was significantly higher in patients treated with morphine and ropivacaine when compared with those injected with morphine alone.

The adverse effects observed in this study were similar to those previously reported. Nausea and vomiting tended to be the most common and were observed in 15.7% (3 of 19) of morphine-only patients and 5.8% (1 of 17) of patients treated with ropivacaine and morphine; these symptoms are frequently reversible with ondansetron. Urinary retention is also relatively common with intrathecal opioids. In our patients, urinary retention often resolved in a few days or weeks, and prolonged bladder dysfunction was not common. Both treatment groups had 1 patient with transient urinary retention. Constipation was not observed, as most patients treated with systemic opioids in this research were treated with a bowel stimulant, a stool softener, or laxatives prior to intrathecal management. Constipation occurred in 1 patient in group R. Somnolence and sedation are rare adverse effects for medullary opioids [25]. We did not observe these effects in our series. Infection, respiratory distress, motor dysfunction, seizures, weight increase, reduced sexual impulses, and port malfunction are also possible risks but were not observed in our series.

Because of the above risks and system implantation costs, proper selection of patients prior to subcutaneous intrathecal port transplantation is critical. The following selection criteria are recommended: (1) great pain despite oral narcotic management or unsatisfactory narcotic side effects at the dosage required to manage pain; (2) medication for the underlying disease, for example, radiation tumor treatment or surgical tumor treatment, that has not caused the pain; and (3) neuroablative processes for pain reduction that were refused by patients or deemed as unsatisfactory by physicians. In addition, the outcome with the intrathecal port is greatly determined by the attitude of the patient and family; a peaceful and friendly environment is important. The implantation of intrathecal catheters connected to subcutaneous injection ports was appropriately indicated for patients in this study on the grounds of life expectancy, expense, and drug/dosage requests.

Our study had several limitations. First, the effective sample size was small, as the study included only 36 patients. Second, long-term complications, such as formation of granulomas and intrathecal infection, were not assessed because of the short follow-up period. Third, mild negative effects, such as sedation or pruritus, may have been underestimated, as the patients showed pharmacological adverse effects in a passive manner in the present study. Fourth, the preoperative pharmacological adverse effects of systemic opioids were not available. Thus, it is not clear whether opioid-mediated side effects were decreased by the intrathecal treatment.

## 5. Conclusions

Intrathecal morphine and ropivacaine management in connection with a transplantable subcutaneous port is a safe and efficient approach to provide treatment of intractable cancer pain. The extra intrathecal ropivacaine enhances pain management and increases the quality of life. The usage of intrathecal ropivacaine is therefore deemed to be safe and acceptable. Large-scale prospective random trials are necessary to assess the advantages and safety of intrathecal ropivacaine.

## Figures and Tables

**Table 1:** Demographics and clinical data^*∗*^.

	Morphine alone	Morphine + ropivacaine
Age (years)	64.0 ± 13.4	62.6 ± 9.92
Sex (male/female)	11/8	14/3
Cancer type (*N*)		
Bladder	2	1
Bile duct	1	0
Colon	2	1
Duodenum	1	1
Esophagus	1	2
Kidney	0	1
Liver	1	2
Lung	3	4
Pancreas	1	0
Penis	1	0
Sarcoma	1	0
Stomach	2	3
Ureter	2	1
Uterus	1	1
Location of pain (*N*)		
Chest	2	3
Abdomen	7	6
Lower limb Preoperative systemic opioid type (*N*)	10	8
Morphine	4	2
Oxycodone	10	11
Fentanyl	5	4

^*∗*^Data from 36 patients.
